# Early Peanut Introduction for Allergy Prevention: Evidence and Practical Implementation in Pediatric Practice

**DOI:** 10.7759/cureus.111378

**Published:** 2026-06-23

**Authors:** Delete Ebere-Bank, Kenneth Soyemi, Rosibell Arcia-Diaz

**Affiliations:** 1 Department of Pediatrics, John H. Stroger, Jr. Hospital of Cook County, Chicago, USA

**Keywords:** clinical practice guidelines, early allergen introduction, eczema, food allergy prevention, immune tolerance, infant feeding, leap trial, peanut allergy, pediatric allergy, risk stratification

## Abstract

Peanut allergy is one of the most common and potentially life-threatening food allergies in children. It frequently persists into adulthood and imposes significant burdens on affected families, including dietary vigilance, social restrictions, and the constant risk of anaphylaxis. For many years, clinical guidelines recommended delaying the introduction of allergenic foods during infancy, based on the assumption that limiting early exposure would reduce the risk of sensitization and subsequent development of allergy. However, accumulating epidemiological observations and rigorous clinical trial data have fundamentally reversed this paradigm, demonstrating that early oral exposure to peanut protein during a critical window in infancy promotes immune tolerance rather than sensitization.

This narrative review synthesizes the key evidence underpinning this paradigm shift, beginning with the epidemiological observations that prompted investigation and centering on the landmark Learning Early About Peanut Allergy randomized controlled trial, which demonstrated a substantial relative risk reduction in peanut allergy among high-risk infants who consumed peanut regularly during early infancy through early childhood. The review also examines long-term follow-up data through adolescence confirming the durability of this protective effect, supporting evidence from the Enquiring About Tolerance trial and pooled analyses, and the biological rationale for early oral tolerance induction. Finally, it provides practical, risk-stratified guidance aligned with current recommendations from the National Institute of Allergy and Infectious Diseases, the American Academy of Pediatrics, and other major medical organizations, addressing clinical implementation strategies, safety considerations, and barriers to adoption in routine pediatric care.

## Introduction and background

Peanut allergy has become an important health concern, especially in developed countries, where it affects between 1% and 4.5% of children [1,2]. Peanut allergy is among the most common causes of severe allergic reactions and creates lasting burdens for families, including constant ingredient monitoring, social limits, and persistent anxiety [1]. For years, guidelines advised parents to delay the introduction of peanuts in young children, especially those at higher risk due to moderate-to-severe eczema, egg allergy, or other food allergies [3].

Clinicians once viewed avoidance as logical because they believed that limiting exposure would prevent the development of allergies. Later evidence showed that early peanut exposure may instead promote immune tolerance, the ability of the immune system to recognize and accept food proteins without mounting an allergic response [4,5]. One pivotal observation found that Jewish children in the United Kingdom had peanut allergy rates 10 times higher than Jewish children in Israel, despite similar genetic backgrounds. Israeli infants commonly ate peanut-containing snacks by about seven months of age, while British infants usually avoided peanuts during the first year [1,6].

This observation prompted a major reassessment of allergy prevention. Infants with moderate-to-severe eczema and/or egg allergy are considered at risk for peanut allergy because of their underlying atopic predisposition and impaired skin barrier function, which may facilitate allergic sensitization [2,7]. This article reviews the evidence supporting early peanut introduction and describes practical strategies for using this approach in routine pediatric care.

To inform this narrative review, literature was identified through targeted searches of PubMed and relevant guideline documents from professional organizations, including the National Institute of Allergy and Infectious Diseases (NIAID) and the American Academy of Pediatrics (AAP). Search terms included "early allergy prevention", "peanut allergy prevention", "food allergy prevention", "LEAP trial", and related keywords. Additional relevant studies were identified through review of reference lists from landmark trials, guideline statements, and review articles. Landmark clinical trials, guideline statements, pooled analyses, and relevant review articles were selected based on their relevance to the prevention of peanut allergy and the practical implications of early peanut introduction strategies.

## Review

The LEAP trial: a paradigm shift

The Learning Early About Peanut Allergy (LEAP) trial, published in 2015, fundamentally transformed the approach to peanut allergy prevention and stands as one of the most influential randomized controlled trials in pediatric allergy [1,4,5]. The trial was directly inspired by the striking epidemiological observation that peanut allergy prevalence differed 10-fold between Jewish children in the United Kingdom and Israel, populations with similar genetic backgrounds but markedly different infant feeding practices [1,6]. This cross-cultural disparity suggested that early dietary exposure to peanut might protect against allergy development rather than promote it, directly contradicting the prevailing avoidance-based paradigm.

The LEAP trial enrolled 640 infants aged 4-11 months who were at high risk for peanut allergy because they had severe eczema, egg allergy, or both [1,5]. These inclusion criteria were chosen because severe eczema and egg allergy are the strongest known risk factors for the development of peanut allergy, and targeting this population maximized the likelihood of detecting a preventive effect [1,5]. Before enrollment, researchers performed skin prick testing (SPT) to peanut and excluded infants with established peanut allergy, defined as a wheal size of 5 mm or greater, as these infants were considered too likely to have a clinically significant allergy to safely participate in a consumption arm [1,6].

The remaining eligible infants were stratified into two cohorts based on their baseline SPT results: those with no measurable wheal (SPT-negative), representing infants without detectable peanut sensitization, and those with a small reaction measuring 1-4 mm (SPT-positive), representing infants with early, low-level sensitization who had not yet developed clinical allergy [1,6]. This stratification was a critical design feature, as it allowed the investigators to assess whether early peanut introduction could prevent allergy not only in unsensitized infants but also in those who already showed immunological evidence of early sensitization. Within each stratum, infants were randomly assigned to one of two groups: regular consumption of peanut-containing foods or complete avoidance of peanuts until age five [1,5].

Infants assigned to the peanut consumption group ate at least 6 g of peanut protein each week, divided across at least three servings, a dose chosen to approximate the typical peanut intake observed among Israeli infants [1,6]. Adherence was monitored through dietary questionnaires and food diaries. At age five, all children underwent a double-blind, placebo-controlled oral food challenge, the gold standard for diagnosing food allergy, ensuring that the primary outcome was assessed with the highest possible diagnostic accuracy [1,5].

Results

The results were striking and exceeded expectations in both magnitude and consistency across subgroups. Among the 530 participants in the intention-to-treat population with negative baseline skin prick tests, only 1.9% of those who regularly consumed peanut developed allergy by age five, compared with 13.7% of those who avoided peanut (p = 0.001) [1,6]. This represented an 86.1% relative risk (RR) reduction and a 12.6% absolute risk reduction. The number needed to treat was 8.5, meaning early peanut introduction in about eight to nine high-risk infants prevented one case of peanut allergy [1,6]. These numbers are exceptionally favorable for a preventive intervention and compare well with many established public health measures.

Even among the 98 children with early peanut sensitivity shown by a small positive skin test at baseline, regular peanut intake sharply reduced allergy rates, from 35.3% in the avoidance group to 10.6% in the consumption group (p = 0.004) [1,6]. This represented a 70% RR reduction and a 24.7% absolute risk reduction. In this more sensitized group, the number needed to treat was only four, meaning that treating just four already-sensitized infants with early peanut consumption prevented one case of clinical peanut allergy [1,6]. The finding that early introduction was effective even in infants who already showed immunological evidence of sensitization was particularly important, as it demonstrated that the window for tolerance induction had not yet closed in these children and that early sensitization does not inevitably progress to clinical allergy.

The safety profile of the intervention was reassuring. Among 272 SPT-negative infants who received a baseline oral peanut challenge, only one infant had a moderate reaction consisting of an erythematous urticarial rash, which was successfully treated with an antihistamine [6]. No reactions required epinephrine, and no serious adverse events were attributed to peanut consumption during the trial [1,6]. This safety record was critical for establishing the feasibility of early peanut introduction as a population-level strategy.

Long-term follow-up

A key question following the LEAP trial was whether the protective effect of early peanut introduction would persist after peanut consumption ceased, or whether it represented only temporary desensitization requiring ongoing exposure. The LEAP-On study was designed to address this question directly [1]. In LEAP-On, researchers asked children who had consumed peanuts in the original trial to stop eating peanuts entirely for one year [1]. This avoidance period tested whether the immune tolerance induced by early consumption was durable or would wane without continued dietary exposure.

When tested again at age six after the year of avoidance, the peanut-consumption group still had a 74% lower peanut allergy rate than children who had avoided peanuts from the start [1]. This finding was highly significant because it demonstrated that the protective effect of early introduction was not dependent on continuous peanut intake [1,6]. Rather, early oral exposure during a critical developmental window appeared to establish lasting immunological tolerance that persisted even after a prolonged period without peanut consumption [1].

The LEAP-Trio study extended these findings further by following participants to age 13 years [7]. The cumulative protective effect of early peanut introduction remained at 75% reduction in peanut allergy in the intention-to-treat analysis, reflecting that 10 children would need to introduce early peanut consumption to prevent one case of peanut allergy (95% CI, 6-17) [7]. Only one of the 255 children in the LEAP-Trio from the original consumption group developed peanut allergy between ages 6 and 13 years [7]. Children in the original consumption group had smaller peanut skin-prick test wheal sizes, lower Ara h2-specific IgE levels, higher peanut-specific IgG4 levels, and a higher IgG4:IgE ratio compared with the original avoidance group [7]. These findings demonstrate that early allergen exposure has a long-term influence on immune biomarkers, irrespective of consumption patterns in the school-age years [7]. The durability of this effect has important practical implications, as it suggests that families do not need to maintain rigid peanut consumption schedules indefinitely to preserve the benefit of early introduction.

Supporting evidence from other trials

The Enquiring About Tolerance (EAT) trial complemented the LEAP findings by testing a broader allergen introduction strategy in a general, unselected population [8,9]. Unlike LEAP, which focused exclusively on high-risk infants, EAT enrolled breastfed infants from the general population regardless of their baseline allergy risk and randomized them to early introduction of six allergenic foods, including peanut, egg, cow's milk, sesame, whitefish, and wheat, beginning at three months of age, vs. standard introduction following current UK guidelines at approximately six months [8,9].

The intention-to-treat analysis did not show a statistically significant benefit for the early-introduction group as a whole [8]. However, this result must be interpreted in the context of the substantial adherence challenges encountered in the trial. Only 42% of participants in the early-introduction group successfully followed the full protocol, which required introducing and maintaining regular consumption of six different allergenic foods simultaneously beginning at three months of age [8,9]. Many infants were likely not developmentally ready to accept complementary foods at this early age, and some parents reported significant feeding difficulties, including infant refusal, gagging, and the logistical burden of preparing and administering multiple allergens on a regular schedule [3,8,9].

In the per-protocol analysis of participants who successfully followed the early-introduction plan, the protective effect was substantial and statistically significant. No children in the early-introduction group developed peanut allergy, compared with 2.5% in the standard-introduction group (p = 0.003) [8]. Dose-response analysis further showed that consuming at least 2 g of peanut protein per week was associated with a significantly lower peanut allergy prevalence, suggesting a threshold effect for the protective benefit [8]. The divergence between the intention-to-treat and per-protocol results highlights the practical challenges of implementing early allergen introduction strategies, particularly when families are asked to introduce multiple allergens simultaneously. It also underscores the importance of developing feasible, family-centered approaches that maximize adherence in real-world settings.

The PreventADALL trial provided additional supporting evidence, demonstrating that introduction of allergenic foods before age four months reduced food allergy in early childhood in a general population, with a significant effect for peanut allergy specifically [10]. These findings, obtained in the absence of screening for risk of atopic disease, further support the broader applicability of early introduction strategies beyond high-risk populations [10].

Pooled and causal inference analyses

Pooled analyses combining individual participant data from the LEAP and EAT trials provided additional statistical power and allowed examination of the effect across a broader range of risk profiles [11]. These analyses demonstrated a 75% reduction in peanut allergy among children randomized to consume peanut from early infancy (p = 0.0001) [11]. Importantly, this benefit appeared consistent across all levels of eczema severity, from no eczema to severe eczema, and across different ethnic groups. This finding was critical because it extended the evidence base beyond the high-risk population studied in LEAP and suggested that early peanut introduction has preventive value across the full spectrum of allergy risk, not only among the highest-risk infants [11].

Causal inference analyses within these pooled data further strengthened the conclusion that the relationship between early peanut introduction and reduced allergy risk is causal rather than merely associational [11]. In the pooled per-protocol analysis, peanut consumption reduced the risk of peanut allergy by 98% (p = 0.0001), and a multivariable causal inference approach estimated a 100% reduction in peanut allergy among children without eczema (p = 0.004) [11]. These analyses accounted for potential confounders and selection biases, providing additional confidence that the observed protective effect is directly attributable to early peanut consumption. Earlier age of introduction was associated with improved effectiveness of the intervention [11].

Interpretation of evidence

The LEAP trial provides high-quality Level I evidence that early peanut introduction significantly reduces peanut allergy risk in high-risk infants [1,4,5]. The benefit is large and clinically meaningful, and the use of double-blind, placebo-controlled oral food challenges as the primary outcome measure strengthens the reliability and validity of the results [1,5]. The consistency of findings across the SPT-negative and SPT-positive strata, the durability demonstrated in LEAP-On and LEAP-Trio, and the confirmatory per-protocol results from EAT collectively build a robust evidence base [1,7,8].

Meta-analyses incorporating data from multiple studies confirm that introducing peanuts during infancy reduces the risk of peanut allergy. A systematic review and meta-analysis of four trials (3,796 participants) showed high-certainty evidence that earlier introduction of peanut between 3 and 10 months of age was associated with decreased risk of peanut allergy (RR, 0.31; 95% CI, 0.19-0.51; I² = 21%) [11]. In a population with 2.5% peanut allergy prevalence, this translates to preventing approximately 17-18 peanut allergy cases per 1,000 individuals [1,11]. These numbers underscore the substantial public health potential of early peanut introduction as a population-level preventive strategy.

Although LEAP focused on high-risk infants, subsequent research, pooled analyses, and expert guidelines support broader application of early peanut introduction across all risk groups [3,11]. Population modeling has further refined the understanding of optimal timing and emphasizes that earlier introduction offers greater protection [12]. Evidence supports a critical window between four and six months of age, during which the developing immune system may be most receptive to oral tolerance induction [1,12]. The estimated reduction in peanut allergy diminishes with each month of delayed introduction. If introduction is delayed until 12 months of age, the estimated risk reduction falls to only 33%, compared with the much larger reductions observed with introduction at four to six months [12]. These modeling estimates assume perfect adherence and implementation, which may not reflect real-world practice, but they nonetheless highlight the importance of timely introduction [12].

Biological rationale

The biological explanation for the protective effect of early peanut introduction centers on the concept of oral immune tolerance [4,5,13]. The developing infant immune system is thought to pass through a critical window during which oral exposure to food proteins promotes the development of regulatory immune responses that suppress allergic sensitization [11,13]. Early and regular oral exposure to peanut protein during this window appears to activate tolerogenic pathways in the gut-associated lymphoid tissue, leading to the generation of regulatory T cells and other immune mechanisms that establish long-lasting tolerance to peanut antigens [11,13]. Immunological data from the LEAP cohort demonstrate that early peanut consumption diverts the immune response toward a presumably protective effect, with consumers generating peanut-specific IgG4 earlier and in greater quantities than nonconsumers, while only avoiders tend to generate significant quantities of sequential epitope-specific IgE [11].

Conversely, delayed introduction may increase the risk of allergy through an alternative sensitization pathway [4,14,15]. In infants with eczema and impaired skin barrier function, environmental peanut protein can penetrate the disrupted skin and encounter cutaneous immune cells that promote allergic sensitization rather than tolerance [14,15]. This dual-exposure hypothesis proposes that the route of first exposure determines the immune outcome: oral exposure promotes tolerance, while cutaneous exposure through damaged skin promotes sensitization [14,15]. By introducing peanut orally before significant cutaneous sensitization occurs, early dietary exposure may preempt the allergic pathway and establish tolerance as the dominant immune response [4,14].

It is important to note that the protective effect of early peanut introduction is allergen-specific [16]. Early peanut consumption reduces the risk of peanut allergy but does not prevent eczema, asthma, or allergies to other foods [16]. Each allergenic food may require its own early introduction strategy, and the optimal timing and dose for other allergens remain areas of active investigation [1,3,11].

Clinical application

Major medical organizations, including the NIAID, the AAP, and the American Academy of Allergy, Asthma, and Immunology, have translated this evidence into formal clinical practice guidelines [3,6,17]. These guidelines represent a consensus across multiple specialty societies and have been endorsed by numerous pediatric and allergy organizations worldwide [3,6,17,18]. Their publication marked a historic reversal of decades of avoidance-based recommendations and established early peanut introduction as the new standard of care for peanut allergy prevention [18].

Risk-stratified approach

Current guidelines recommend a three-tiered, risk-stratified approach that tailors the timing, setting, and need for prior testing to each infant's allergy risk level [3,6,17].

High-Risk Infants (Severe Eczema and/or Egg Allergy)

These infants carry the greatest risk of developing peanut allergy and should begin peanut-containing foods around four to six months of age [1,6]. However, because of their elevated risk, clinicians should perform allergy testing before introduction [1,6]. The NIAID guidelines define three testing categories based on skin prick test results [6].

SPT ≤2 mm or peanut-specific IgE 0.35 kUA/L: These results indicate minimal or no sensitization. Peanut can be introduced at home with a cumulative first dose of about 2 g of peanut protein. If caregivers or clinicians have concerns about the safety of home introduction, supervised office feeding can be offered as an alternative to provide additional reassurance [6].

SPT 3-7 mm: These results indicate possible early sensitization, and the infant may or may not be clinically allergic. A supervised peanut feeding or graded oral food challenge should occur in a specialist's office or a facility equipped to manage allergic reactions. Infants in this group may be sensitized without being truly allergic and may benefit substantially from early consumption if the supervised feeding produces no reaction [6].

SPT ≥8 mm: These results indicate a high likelihood of established peanut allergy. These infants should be referred for specialist evaluation and management [6].

Moderate-Risk Infants (Mild-to-Moderate Eczema)

These infants have a moderately elevated risk of peanut allergy but do not require allergy testing before introduction. Peanut-containing foods can usually be introduced around six months of age at home without prior testing, based on family preferences and cultural practices [3,6,17].

Low-risk infants (no eczema or food allergy): These infants are at population-level risk for peanut allergy and require no special precautions beyond standard feeding safety. Peanut-containing foods can be introduced freely alongside other solid foods when the infant is developmentally ready, based on family preferences and cultural practices [3,6,17].

Practical considerations

Safety remains essential when implementing early peanut introduction. Caregivers should introduce peanuts in developmentally appropriate, infant-safe forms to minimize the risk of choking [6]. Appropriate preparations include smooth peanut butter thinned with water, breast milk, or formula to a consistency that is easy for infants to swallow; peanut flour or peanut powder mixed into purees, cereals, or other soft foods; and peanut puff snacks softened with liquid until they dissolve easily [6]. Whole peanuts, peanut pieces, and chunky peanut butter are choking hazards and should be strictly avoided in infants and young children.

The safety data from the LEAP trial are reassuring for clinical practice. Among 272 SPT-negative infants who received a baseline oral peanut challenge, only one infant had a moderate reaction, an erythematous urticarial rash that was successfully treated with an antihistamine [1,6]. No reactions required epinephrine, and no serious adverse events were attributed to peanut consumption throughout the trial period [1,6]. This favorable safety profile supports the feasibility of early peanut introduction as a routine preventive measure, even in high-risk infants, when appropriate screening and precautions are followed.

Parental education and reassurance are critical components of successful implementation [1,18]. Parents often need direct, empathetic communication about the safety and preventive value of early peanut introduction, particularly given that many parents of young children today grew up during the era of avoidance recommendations and may have deeply ingrained concerns about introducing allergenic foods early. Clinicians should proactively address parental anxiety, as fear of allergic reactions remains one of the most significant barriers to implementation [1,18]. Providing clear instructions on appropriate peanut preparations, expected versus concerning symptoms, and when to seek medical attention can help build parental confidence.

The recommended maintenance intake is approximately 6-7 g of peanut protein per week, divided across three or more feedings, consistent with the dose used in the LEAP trial [1,6]. Adherence data from the LEAP trial showed that at 12 and 24 months, 75% of children in the peanut-consumption group reported eating at least this amount, suggesting that this dose is achievable for most families [1,6]. Clinicians should encourage families to incorporate peanut into the infant's regular diet in a way that fits naturally with their feeding routines and cultural food practices.

Impact on patient care

Early peanut introduction represents one of the most important and evidence-based changes in pediatric preventive care in recent decades and carries major public health implications [1,4,5]. The reversal from avoidance to early introduction represents a complete paradigm shift in clinical practice [18]. Pediatricians who previously recommended delaying allergenic foods and advising high-risk infants to avoid peanuts until age three now recommend early introduction of peanuts around four to six months of age, using a risk-based approach integrated into routine well-child care [3,6,17].

The potential population-level impact is substantial. A 2025 US study using electronic health record data from the AAP Collaborative Electronic Reporting Network found that the cumulative incidence and risk of developing peanut IgE-mediated food allergy decreased significantly from the preguidelines to postguidelines period (0.79%-0.53%; hazard ratio, 0.65; 95% CI, 0.55-0.77; p = 0.0001), with further reductions in the postaddendum guidelines period (0.45%; hazard ratio, 0.55; 95% CI, 0.46-0.66). Population modeling suggests that peanut allergy prevalence may fall by as much as 77% when all infants receive peanut in the diet, at four months for those with eczema and at six months for those without eczema [12]. This estimate reflects an ideal scenario with perfect adherence and universal implementation, and real-world reductions would likely be somewhat smaller, but even partial implementation could yield meaningful public health benefits [12].

Limitations and future directions

Despite the strength of the evidence supporting early peanut introduction, several important limitations must be acknowledged. The primary LEAP trial enrolled only high-risk infants with severe eczema and/or egg allergy, and while pooled analyses and the EAT per-protocol results support broader application, direct randomized controlled trial evidence in low-risk, general populations remains more limited [6,8,11]. The generalizability of the LEAP findings to populations with different genetic backgrounds, dietary patterns, and environmental exposures also warrants further study, although the pooled analyses showing consistent benefits across ethnic groups are encouraging [11].

Real-world adherence is a significant challenge, as demonstrated by the EAT trial, in which only 42% of participants in the early-introduction group achieved per-protocol adherence [8,9]. Barriers to adherence included developmental readiness for complementary foods at three months of age, infant feeding difficulties such as refusal and gagging, the logistical burden of introducing multiple allergens simultaneously, and parental concerns about allergic reactions [3,8,9]. These findings highlight the gap between the efficacy demonstrated in controlled trial settings and the effectiveness achievable in routine clinical practice.

Implementation barriers persist in many healthcare settings [1,18]. These include limited access to allergy specialists for high-risk infants who require evaluation and testing before peanut introduction; parental anxiety about allergic reactions, which may lead families to delay or avoid introduction despite clinician recommendations; and clinician unfamiliarity with current guidelines, particularly among providers who trained during the avoidance era [1,18]. Cultural feeding practices and family food preferences may also affect uptake, making clear, culturally sensitive, and individualized clinician communication essential for successful implementation [1,3].

Promising strategies to improve guideline adherence and implementation have been developed and validated. These include electronic medical record-integrated clinical decision support tools that prompt clinicians to discuss early peanut introduction during well-child visits, multifaceted educational interventions targeting both clinicians and families, and community-based outreach programs designed to reach underserved populations with limited access to allergy specialists.

Future research should focus on several key areas: evaluating long-term outcomes of early peanut introduction across diverse populations and geographic settings; defining the optimal dose, frequency, and duration of peanut consumption needed to establish and maintain tolerance; and developing strategies to overcome implementation barriers in underserved communities with limited access to specialty care [1,3,11]. Similar early-introduction approaches are under investigation for other common allergenic foods, and positive results from these studies may further transform the landscape of food allergy prevention [1,3,11].

## Conclusions

Early introduction of peanuts during infancy is a safe and highly effective strategy for preventing peanut allergy. High-quality evidence, anchored by the LEAP randomized controlled trial and supported by long-term follow-up data through adolescence, the EAT trial, pooled analyses, and meta-analyses, demonstrates a substantial and durable risk reduction, especially among high-risk infants, with growing support for benefits across all risk groups. Integrating early peanut introduction into routine pediatric care represents a major advance in preventive medicine and may significantly reduce the population burden of food allergy. Clinicians should work with parents to introduce peanut products into infant diets at four to six months of age, following the risk-stratified approach outlined in current clinical practice guidelines.
